# Autophagy at the Crossroads of Protein and RNA Toxicity in Repeat Expansion Cerebellar Ataxias

**DOI:** 10.3390/cells15151411

**Published:** 2026-08-04

**Authors:** Silvia Tortoriello, Simona Rossi, Ilaria Della Valle, Nadia D’Ambrosi, Mauro Cozzolino

**Affiliations:** 1 Institute of Translational Pharmacology, National Research Council (CNR), 00133 Rome, Italy; simona.rossi@ift.cnr.it; 2 Department of Biology, University of Rome Tor Vergata, 00133 Rome, Italy; ilaria.della.valle@uniroma2.it (I.D.V.); nadia.dambrosi@uniroma2.it (N.D.); 3 Graduate Program in Cell and Molecular Biology, Department of Biology, University of Rome Tor Vergata, 00133 Rome, Italy

**Keywords:** autophagy, spinocerebellar ataxia, repeat expansion disorders, RNA toxicity, RAN translation

## Abstract

Repeat expansion cerebellar ataxias comprise a genetically and mechanistically heterogeneous group of neurodegenerative disorders unified by the pathological expansion of short tandem repeats (STRs) beyond a disease-causing threshold. Depending on their genomic localization, these expansions can lead to toxic protein gain-of-function, as in polyglutamine (polyQ) cerebellar ataxias, or to RNA-mediated toxicity and repeat-associated non-AUG (RAN) translation, for which recent evidence supports a major pathogenic role in non-coding spinocerebellar ataxias (SCAs). Despite these distinct upstream mechanisms, disruption of neuronal homeostasis occurs through converging pathogenic processes, including proteostasis impairment, transcriptional dysregulation, and mitochondrial dysfunction, leading to progressive neuronal loss. Importantly, impaired autophagy has been consistently reported across multiple repeat expansion ataxias, including both dominant SCAs and recessive conditions, such as Friedreich’s ataxia, suggesting that impairment of this pathway may represent a shared downstream event in disease progression. Indeed, in polyQ cerebella ataxias, the accumulation of misfolded and aggregation-prone proteins places a substantial burden on cellular quality control systems, particularly the ubiquitin-proteasome system and autophagy. Similarly, in non-coding SCAs, toxic RNA species and RAN-derived peptides might interfere with protein clearance mechanisms and contribute to cellular stress. In this review, we will discuss the evidence supporting autophagy impairment as a convergent pathogenic pathway in repeat expansion cerebellar ataxias.

## 1. Introduction

### 1.1. Repeat-Expansion-Associated Cerebellar Ataxias

Repeat expansion disorders are a heterogeneous group of diseases caused by a shared genetic alteration: the repetition beyond a pathogenic threshold (repeat expansion) of short tandem repeats (STRs, also known as microsatellites) within specific genes [1]. STRs are polymorphic repeats of short nucleotide sequences (up to six base pairs in tandem) widely distributed across both coding and non-coding regions of the human genome, accounting for approximately 3% of its content. In physiological conditions, STRs can act as regulatory elements, modulating DNA methylation, transcription, and alternative splicing [2]. However, due to their intrinsic instability STRs are prone to aberrant repetition that, once expanded beyond their physiological length, induce structural alterations in DNA and trigger a range of molecular mechanisms of RNA or protein gain/loss-of-function, ultimately creating a toxic cellular environment that drives disease onset. Despite sharing distinctive molecular features, including genetic instability, strong correlation between repeat length and disease severity, and anticipation (earlier onset in successive generations), repeat expansion disorders exhibit diverse pathogenic mechanisms depending on the genomic location of the repeat and its specific pathological threshold, resulting in highly heterogeneous clinical manifestation [3]. Expansions within coding regions typically lead to the production of toxic proteins, such as polyglutamine (polyQ) tracts, whereas non-coding expansions are associated to transcriptional silencing, RNA toxicity, formation of nuclear RNA foci, and repeat-associated non-AUG (RAN) translation [4]. Collectively, these disorders predominantly affect the nervous system and are characterized by progressive neurodegeneration, high clinical variability, and a significant disease burden, with an estimated prevalence of approximately 1 in 3000 individuals worldwide [5].

**Figure 1:**
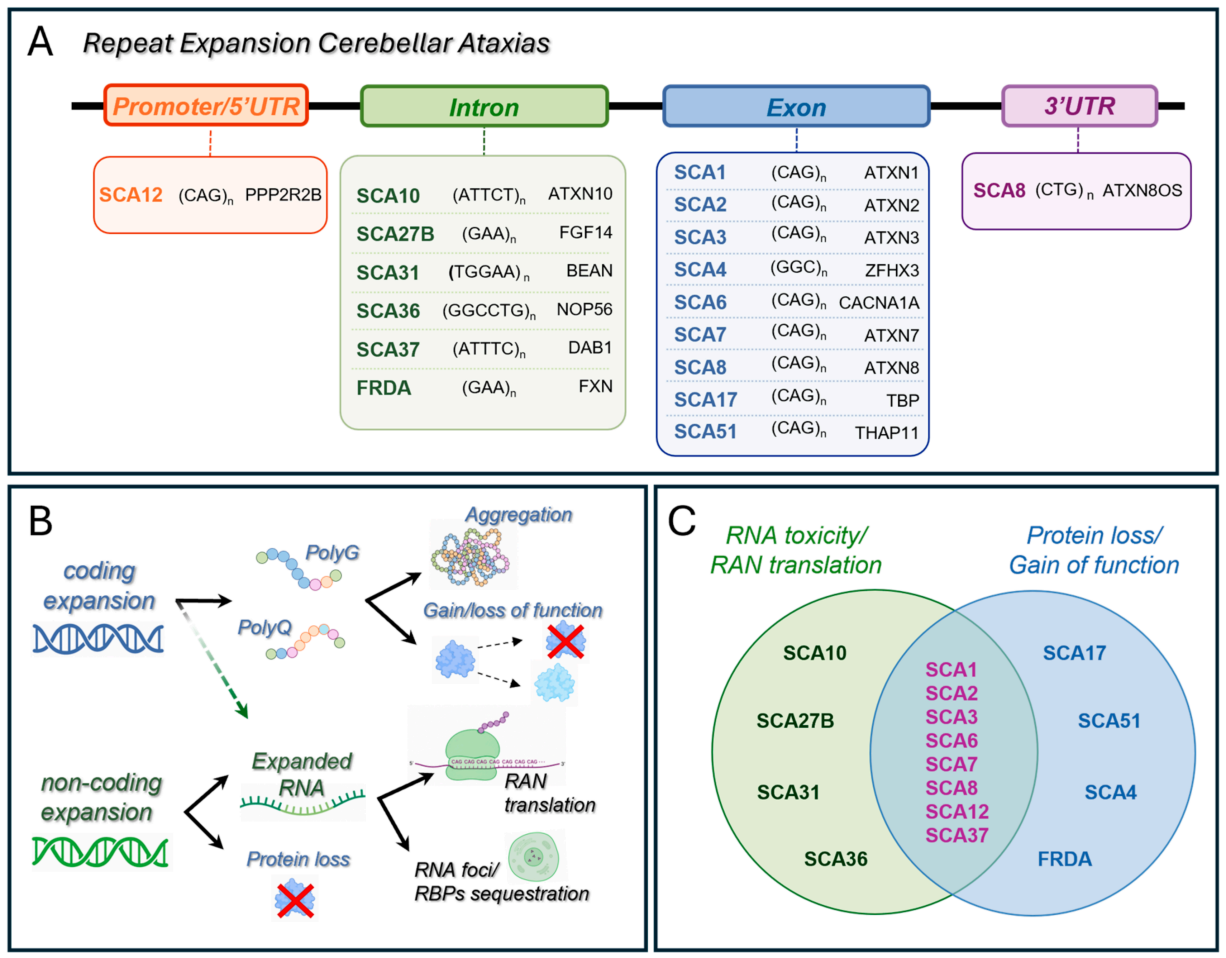
**Genetic and mechanistic landscape of repeat expansion cerebellar ataxias.** (**A**) Schematic overview of repeat expansion mutations across genomic regions and their associated cerebellar ataxias. Expansions can occur in promoter/5′UTR, intronic, exonic, or 3′UTR regions, involving different repeat motifs and associated genes. Coding (exonic) expansions are primarily represented by CAG repeats, while non-coding expansions display greater sequence variability. (**B**) Distinct pathogenic mechanisms associated with coding and non-coding repeat expansions. Coding expansions lead to the production of proteins with elongated polyglutamine (polyQ) or polyglycine (polyG) tracts that undergo misfolding, aggregation, and toxic gain- and/or loss-of-function effects. Non-coding expansions give rise to expanded RNA species that can induce toxicity through RNA-mediated mechanisms and repeat-associated non-AUG (RAN) translation, generating aggregation-prone peptides, but they can also result in protein loss-of-function. (**C**) Overlapping pathogenic mechanisms across cerebellar ataxias. Venn diagram illustrates the contribution of RNA toxicity/RAN translation and protein loss/gain-of-function across different cerebellar ataxia subtypes, highlighting shared and distinct molecular features.

Among repeat expansion disorders, spinocerebellar ataxias (SCAs) represent a clinically and genetically heterogeneous group of autosomal dominant neurodegenerative diseases characterized by progressive cerebellar ataxia, impaired coordination, dysarthria, and oculomotor abnormalities. Beyond cerebellar dysfunction, many SCA subtypes present with additional neurological features, including pyramidal signs, peripheral neuropathy, parkinsonism, cognitive impairment, and retinal degeneration, reflecting widespread neurodegeneration beyond the cerebellum. To date, more than 40 SCA subtypes have been identified, with considerable variability in age of onset, disease progression, and clinical severity. Epidemiologically, SCAs are rare disorders, with a combined prevalence estimated at approximately 1–5 per 100,000 individuals worldwide, although this varies significantly depending on geographic region and population-specific founder effects [6]. From a molecular perspective, SCAs can be clustered depending on the nature and location of the causative mutation. The majority of SCAs are caused by repeat expansion mutations located either in coding or non-coding regions (Figure 1), while a minority (SCA5, SCA13, and SCA14) arise from conventional point mutations.

In SCA1, SCA2, SCA3/MJD, SCA6, SCA7, and SCA17, repeat expansions of the CAG trinucleotide occur in a coding region of the gene of interest and, therefore, encode elongated polyQ tracts that promote protein misfolding, oligomerization, and aggregation. Together with Huntington’s disease (HD), dentatorubral-pallidoluysian atrophy (DRPLA) and spinal and bulbar muscular atrophy (SBMA), which share the same pathogenic mechanism, they are collectively known as polyQ diseases [7]. At the cellular level, polyQ stretches form intranuclear or cytoplasmic inclusions that sequester transcription factors, chaperones, and components of the protein quality control machinery, including elements of the ubiquitin-proteasome system and autophagy pathways. This leads to widespread disruption of proteostasis, transcriptional dysregulation, mitochondrial dysfunction, and ultimately neuronal death. In contrast, non-coding SCAs arise from repeat expansions located in untranslated regions of the associated genes. Although the underlying pathogenic mechanisms have not yet been fully elucidated, current evidence suggests that these disorders are primarily driven by RNA gain-of-function toxicity, as observed in SCA8, SCA10, SCA31, SCA36, and SCA37. Indeed, expanded repeat transcripts can form stable secondary structures and accumulate as nuclear RNA foci that sequester RNA-binding proteins and other factors, thereby disrupting RNA metabolism. Additionally, repeat transcripts may undergo RAN translation, which generates toxic peptides in multiple reading frames, further contributing to neuronal toxicity [8]. Nevertheless, protein loss-of-function mechanisms resulting from repeat-induced transcriptional silencing may also contribute to the pathogenesis of non-coding ataxias. This is notably the case for the autosomal recessive Friedreich’s ataxia (FA), which is caused by a GAA repeat expansion within a non-coding region of the *frataxin* (*FXN*) gene, that leads to reduced frataxin expression and cerebellar dysfunction. FA is the most frequent inherited ataxia, with an average prevalence of 0.5 cases per 100,000 individuals. Although progressive loss of balance and coordination represent its characteristic feature, FA is being increasingly recognized as a multi-system disorder, since patients can present with extra-neural comorbidities, including cardiomyopathy, musculoskeletal defects, and metabolic dysfunctions [9].

Despite genetic and mechanistic diversity among SCAs, increasing evidence suggests that both protein aggregation in polyQ SCAs and RNA-mediated toxicity in non-coding SCAs may ultimately converge on common cellular pathways, including defects in intracellular degradation systems. This convergence highlights the potential importance of autophagy not only in the clearance of cytosolic aggregates but also in the regulation of cellular homeostasis in the context of repeat expansion disorders. In this review, we summarize current evidence linking autophagy dysfunction to the pathogenesis of coding and non-coding repeat expansion cerebellar ataxias, with particular emphasis on its interplay with protein aggregation, RNA toxicity, and RAN translation. We also discuss the therapeutic potential of autophagy-modulating strategies and the major challenges that remain to be addressed in the field.

### 1.2. Canonical and Selective Autophagy Pathways: An Overview

Autophagy is an evolutionarily conserved lysosomal degradation pathway that maintains cellular homeostasis through the turnover of proteins, protein aggregates, and damaged organelles [10]. Three major forms of autophagy have been described—macroautophagy, microautophagy, and chaperone-mediated autophagy (CMA)—which differ in their mechanisms of cargo delivery to lysosomes [11,12,13]. Comprehensive discussions of autophagy mechanisms can be found in several recent reviews [14,15,16]. Since macroautophagy is the major form implicated in neurodegenerative diseases, including cerebellar ataxias, only this pathway will be briefly outlined here and referred to throughout the manuscript as “autophagy”.

Autophagy involves the sequestration of cytoplasmic cargo within double-membrane autophagosomes that subsequently fuse with lysosomes, where cargo is degraded and recycled [14]. Autophagy is tightly regulated by nutrient- and stress-responsive pathways, particularly mTORC1 signaling, and depends on a coordinated cascade of autophagy-related proteins controlling autophagosome biogenesis, cargo recognition, and lysosomal degradation [17,18,19,20]. During this process, the ubiquitin-like protein LC3 undergoes cleavage to LC3-I and lipidation to LC3-II to become associated with autophagosomal membranes, whereas cargo receptors such as SQSTM1/p62 recognize ubiquitinated substrates and facilitate their delivery to autophagosomes [15]. Given the post-mitotic nature of neurons and their high susceptibility to proteotoxic stress, efficient autophagic clearance is essential for maintaining neuronal homeostasis.

Beyond bulk degradation, autophagy can selectively target specific substrates through selective cargo receptors, including SQSTM1/p62, optineurin (OPTN), and Neighbor of BRCA1 gene 1 (NBR1), that recognize ubiquitinated targets and mediate their delivery to autophagosomes. One of the best-characterized forms is mitophagy, the selective removal of damaged mitochondria, primarily regulated by the PTEN-induced kinase 1 (PINK1)/Parkin pathway. Mitochondrial damage disrupts mitochondrial membrane potential, preventing PINK1 translocation into the inner mitochondrial membrane (IMM), thereby favoring its accumulation on the outer mitochondrial membrane (OMM). Here, PINK1 phosphorylates the ubiquitin chain to recruit Parkin, which acts as an E3 ligase ubiquitinating several OMM proteins, later recognized by ubiquitin-binding mitophagy receptors. Some mitophagy receptors can also recognize hypoxic mitochondria in a ubiquitin-independent manner, directly binding both the cargo and LC3 to promote mitochondria clearance [16].

A crucial substrate of selective autophagy is represented by misfolded or aggregated proteins, which generate potentially cytotoxic inclusions, usually degraded by aggrephagy. SQSTM1/p62 plays a key role in aggrephagy because it binds ubiquitin on misfolded proteins and thus recruits the autophagy machinery. However, SQSTM1/p62 can also bind the ULK1 complex itself to promote autophagosomes’ formation directly at the site of protein aggregates. Details of such interaction are still unknown, but it is believed to depend on the ability of SQSTM1/p62 to undergo self-oligomerization, following polyubiquitin binding, and liquid–liquid phase separation that generates SQSTM1/p62 droplets. These p62 bodies are dynamic and transient structures observed in physiological conditions, but mainly in response to different stress conditions [21]. Furthermore, hetero-oligomerization of SQSTM1/p62 with aggrephagy receptor NBR1 is believed to increase the receptors’ affinity for ubiquitinated proteins, ending in a more efficient process that becomes particularly relevant in neurodegenerative disorders characterized by proteotoxic stress. Downstream of p62/NBR1 interaction, other factors, such as Tax1 Binding Protein 1 (TAX1BP1), are recruited at p62 bodies to promote autophagosome biogenesis [22]. TAX1BP1 was also implicated in the clearance of aggregates formed by mutant Huntingtin (mHtt), the leading cause of HD, in an LC3-independent manner, via direct binding to the ULK1 complex, much like SQSTM1/p62 [23]. Besides canonical aggrephagy mechanisms, in the context of polyQ diseases, TRiC subunit chaperonin-containing TCP-1 subunit 2 (CCT2) has been identified as a ubiquitin-independent player in aggregates’ clearance. Unlike the SQSTM1/p62/NBR1/TAX1BP1 pathway that uses liquid–liquid phase separation to clear protein condensates, CCT2 promotes autophagic removal of solid mHtt aggregates via a direct interaction with LC3 [24].

Although much less characterized, additional forms of selective autophagy have been recently described, and emerging evidence indicates that autophagy also contributes to RNA homeostasis by regulating transport, secretion, and decay of RNAs, RNA binding proteins (RBPs), and ribonucleoprotein (RNP) complexes. Indeed, RNautophagy is an unconventional autophagy pathway that mediates the degradation of RNA species through direct transport to the lysosome. RNA uptake through the lysosomal membrane is mediated by lysosomal factors LAMP2C and SID1 transmembrane family member 2 (SIDT2) [25,26]. As described, RNautophagy resembles CMA, where the HSC70 chaperone recognizes, binds, and transports proteins to the lysosome and, therefore, it has been suggested that this pathway may represent a CMA-like mechanism exploited by mammalian cells to control RNA homeostasis. In stressful conditions, cells can store mRNAs in cytoplasmic membraneless organelles with RBPs generating RNP granules, such as P-bodies and stress granules. Both these organelles are an established autophagy substrate, through a selective mechanism known as granulophagy [27]. However, recent evidence demonstrated a non-canonical function for autophagy factors ULK1/2 in promoting stress granules’ disassembly in an autophagy-independent manner. Additionally, autophagy is known to be involved in the selective clearance of ribosomes, detected inside autophagosomes via electron microscopy. Based on recent evidence, ribophagy may contribute to amino acids and nucleotides’ recycling in physiological conditions and act as a response mechanism to promote survival under ribosomal stress conditions [28]. To further characterize the autophagy–RNA interplay, an increasing number of studies are focusing on the post-transcriptional and translational regulation of autophagy by RBPs and non-coding RNAs, adding much more complexity to the interaction [29].

Overall, selective mechanisms of autophagy highlight the versatility of this pathway as a central node in maintaining both proteostasis and RNA homeostasis. Accordingly, their coordinated dysfunction may, therefore, represent a critical pathogenic node in disorders characterized by both protein aggregation and RNA toxicity, such as spinocerebellar ataxias.

## 2. Proteostasis Failure in Polyglutamine Cerebellar Ataxias: Implications for Autophagy Dysfunction

Repeat expansion mutations occurring in coding regions of the genome typically involve the abnormal expansion of a cytosine-adenine-guanine (CAG) trinucleotide repeat, which—once translated—generates proteins with a distinguished aberrant structure characterized by elongated polyglutamine (polyQ) tracts. These expansions represent the molecular hallmark of a group of neurodegenerative disorders collectively known as polyQ diseases. To date, polyQ disorders include autosomal dominant diseases, such as spinocerebellar ataxias (SCA) types 1, 2, 3, 6, 7, and 17, as well as HD, DRPLA, and X-linked spinal SBMA.

Although not fully characterized, proteins harboring polyQ stretches are known to undergo conformational changes to β-sheet-rich structures that later aggregate into large insoluble fibrillary deposits and accumulate as toxic intracellular inclusions, thereby fostering neurodegeneration. Such transition was recently observed by Zhao and colleagues [30] in mutant huntingtin (mHtt, 64Q, and 150Q), the protein associated to HD. Htt was seen to form amyloid-like fibrils by a liquid-to-solid phase transition, highlighting the existence of an intermediate amorphous aggregate phase that could interact with aggrephagy receptor SQSTM1/p62 and be engulfed by autophagosomes. Instead, when the amorphous polyQ matter shifts its composition to fibrils the nucleating phagophores remain sequestered in their solid structures [30]. These findings not only demonstrate that autophagy is directly involved in polyQ aggregates’ clearance but also suggest that its efficiency depends on the biophysical state of the aggregates. Accordingly, autophagy may represent a promising therapeutic target in polyglutamine diseases, although its modulation must be carefully balanced, as its impairment may lead to progressive accumulation of pathological aggregates, whereas its overstimulation could become toxic for cells [31].

### 2.1. SCA3

SCA3/MJD, the most common SCA worldwide, is caused by CAG expansions in the gene *ATXN3*, producing a mutant ataxin-3 protein prone to misfolding and aggregation. Ataxin-3 is a deubiquitinating enzyme known to act not only as a negative regulator of mTORC1 in response to amino acid deprivation, thereby indirectly activating autophagy, but also to directly modulate the autophagy pathway by stabilizing Beclin-1 through its wild-type polyQ tract. Indeed, in SCA3/MJD patient-derived fibroblasts Beclin-1 is downregulated both at mRNA and protein levels, indicating, in combination with pSQSTM1/p62 accumulation and LC3-I failed lipidation to LC3-II, an impairment in phagophore nucleation, which may lead to defective autophagosomes’ formation. Accordingly, restoration of Beclin-1 levels via overexpression can reverse the autophagic dysfunction phenotype [32]. Ataxin-3 interaction with Beclin-1 is disrupted also in vivo in si*ATXN3* transfected mice, significantly impairing starvation-induced autophagy [33]. Lentivirus-mediated cerebellar overexpression of Beclin-1 in pre- and post-symptomatic lentiviral-based Lv-AtxQ72 mouse models was able to increase the clearance of polyglutamine-expanded aggregates in the Purkinje cell layer of the cerebellar cortex; also, the same Beclin-1 overexpression in the established SCA3/MJD transgenic Tg-Q69 mouse model at the same time point (10 weeks after lentiviral injection) resulted in a less aggregated and more diffused staining of mutated ataxin-3. As a result, mice behavioral performances were improved, regaining motor coordination, balance, and a correct gait [34]. Exploiting a similar genetic approach, the same authors demonstrated that SCA3/MJD mice pathology could be improved by autophagy induction also in a Beclin-1-independent manner. Indeed, in the mouse model of the disease previously used, Unc-51-like kinase 1/2 (ULK1/2) downregulation was assessed: ULK1/2 is a Beclin-1 upstream autophagy initiator and once its levels were restored by adeno-associated virus (AAV) gene transfer, the autophagy flux was reinstated. As a consequence, aberrant ataxin-3 inclusions were significantly reduced, and the volume of both the molecular layer and whole cerebellum increased, as well as the Purkinje cell number; also, mice ameliorated their performances on both stationary and accelerated rotarod and their gait was improved [35]. Although mutant ataxin-3 seems to impair autophagy by destabilizing Beclin-1, as previously discussed, the beneficial effects of ULK1 overexpression suggest that residual Beclin-1 activity may still be sufficient to sustain autophagosome formation when upstream signaling is enhanced. Moreover, ULK1 regulates multiple early steps of autophagy beyond Beclin-1 activation, indicating that strengthening the upstream autophagic machinery may compensate, at least in part, for Beclin-1 dysfunction. Alongside key components of the autophagy pathway, findings on other molecular factors confirmed the crucial involvement of autophagy in this disease. On Tg-Q69 SCA3/MJD mouse models, Duarte-Neves and colleagues [36] exploited lentiviral vectors to overexpress Neuropeptide Y in mouse cerebella. Neuropeptide Y is a 36-amino-acid neuroprotective peptide abundantly present in several brain regions, able to induce autophagy and found downregulated in SCA3 mice. By restoring its expression, protein aggregates in Purkinje cells were reduced, cerebellar volume increased, and SCA3/MJD Tg mice recovered balance and motor coordination, measured, respectively, by static rotarod, beam walking, and footprint pattern [36,37].

From a clinical point of view, autophagy pharmacological modulation is very appealing. Besides canonical autophagy-activating drugs, n-butylidenephthalide (n-BP), extracted from the herb *Angelica sinensis*, was demonstrated to be a positive regulator for autophagy, probably acting via the AKT/mTOR pathway. Zhou and colleagues [38] tested such compound on SOD1^G93A^ mouse models of amyotrophic lateral sclerosis (ALS), a progressive neurodegenerative disease mainly affecting motor neurons. Mice were treated with n-BP from post-natal day 60 (p60) to their death age while recording their hindlimb grip strength and later assessing lifespan, motor neuron survival, and neuroinflammation: mice treated with n-BP showed a better outcome of the disease, comparable if not better than SOD1^G93A^ mice treated with Riluzole, the only FDA-approved drug for ALS [38]. After 5 weeks of treatment with n-BP, 23-week-old SCA3 mice showed a significant improvement in motor performances tested with rotarod, compared to the vehicle-treated mice. Their cerebellar metabolic activity was also improved, as assessed by PET-MRI glucose decrease, while Purkinje cell arborization and number were rescued [39]. Great interest has grown around trehalose, a disaccharide of two glucose molecules that vertebrates are not able to physiologically synthesize but obtain through diet. In different cellular and animal models, trehalose was observed to enhance autophagy in a mTORC1-independent manner, via either transcription factor EB (TFEB) or AMP-activated protein kinase (AMPK) activation. The efficacy of trehalose in clearing protein aggregates has already been established in other neurodegenerative diseases, such as Alzheimer’s diseases and Parkinson’s disease, respectively characterized by Aβ plaques and α-synuclein aggregates, but it also showed promising results in animal models of HD, where Htt is prone to aggregation and accumulation [40]. Consistently, SCA3/MJD-associated neuropathology in both lentiviral-based and transgenic mouse models is reduced by prolonged trehalose treatment [41].

Consistent with polyQ pathology observed in mammalian systems, transgenic SCA3 zebrafish expressing human ATXN3-84Q exhibit accumulation of autophagy substrates, including increased SQSTM1/p62 levels and altered LC3-II/I ratios, indicative of impaired autophagic processing at both larval and advanced disease stages. Importantly, zebrafish models have provided valuable in vivo evidence linking autophagy dysfunction to aggregate formation and motor impairment. Detergent-insoluble ATXN3 aggregates can be detected as early as two days post-fertilization (dpf), before the onset of behavioral deficits, suggesting that protein aggregation represents an early pathogenic event. Pharmacological manipulation of autophagy further supports a causal role for this pathway in disease progression: inhibition of autophagy with chloroquine increases the burden of insoluble ATXN3 species, whereas autophagy induction with calpeptin reduces aggregate accumulation in both cellular and zebrafish models. Notably, calpeptin-mediated aggregate clearance is associated with improved swimming behavior in SCA3 zebrafish, linking restoration of protein quality control mechanisms to functional benefit. Additional studies demonstrated that both mTORC1-dependent (rapamycin) and mTORC1-independent (trehalose and loperamide) autophagy activators reduce aggregate burden and ameliorate motor deficits [42,43]. More recently, sodium butyrate was shown to enhance autophagic activity, decrease insoluble ATXN3 accumulation, and improve locomotor performance, effects that were abolished by co-treatment with the autophagy inhibitor chloroquine [44]. Together, these findings support a causal relationship between restoration of autophagic flux, aggregate clearance, and functional improvement in SCA3, while highlighting the utility of zebrafish models for in vivo investigation of autophagy-targeting therapies.

Interestingly, Han and colleagues [45] tested a cell-therapy-based approach on iPSC-induced neurons carrying mutant ataxin-3. Notably, in SCA3-derived neurons, levels of autophagy proteins Beclin-1 and LC3-II were significantly lower when compared to control-derived neurons, whereas SQSTM1/p62 was found increased, suggesting a recapitulation of the phagophore nucleation defect observed in mice and human fibroblasts; however, such impairment could be rescued by co-cultures with wild-type mesenchymal stem cells (MSCs), which are known to release neurotrophic factors and vesicles, such as exosomes, and are increasingly used in in vitro treatments for neurodegenerative diseases. Furthermore, lower levels of mutated ataxin-3 were observed in the nucleus of SCA3-induced neurons when cultured in combination with MSCs, indicating that autophagy enhancement from MSCs could deplete the pool of polyQ. The pathway implicated in autophagy activation following MSCs exposure involved TFEB dephosphorylation and nuclear translocation via mTORC1 [45]. TFEB contribution to polyQ pathological autophagy mechanisms was also documented by Yang and colleagues [46] in HD, where they demonstrated TFEB ability to co-aggregate with mHtt via a prion-like domain at its N-terminus both in vitro and in vivo. Such evidence might provide an explanation for the capability of TFEB to reduce mHTT soluble fraction, but not its aggregates [46]. Altogether, these observations further support autophagic impairment as a mechanistic feature of SCA3.

### 2.2. SCA2 and SCA6

SCA2 is caused by expanded CAG repeats in the *ATXN2* gene, resulting in an ataxin-2 protein with an extended polyQ tract that forms cytoplasmic inclusions. Such aggregates are clearly detectable post-mortem in patients’ cerebellum, alongside abnormal puncta accumulation of SQSTM1/p62 and LC3B, revealing a perturbation in the autophagy pathway also in SCA2 pathogenesis, that is recapitulated in lentivirus-generated mouse models of the disease (Atx2Q58 and Atx2Q104). Importantly, pharmacological activation of autophagy by cordycepin, a compound acting via the AMPK pathway, was able to reinstate the autophagy flux and, therefore, ameliorate SCA2 neuropathology by reducing aggregate burden in vivo [47]. In patient-derived fibroblasts, mutant ATXN2 accumulates preferentially in the insoluble fraction and is associated with increased oxidative stress and apoptotic activation. Pharmacological inhibition of autophagy further exacerbates these phenotypes, whereas enhancement of autophagy or solubilization of ATXN2 oligomers attenuates cell death, further supporting a protective role of protein clearance mechanisms in SCA2 [48]. Beyond these observations, recent studies have provided mechanistic insights into the interplay between ATXN2 and autophagy. STAU1, an RNA-binding protein involved in stress granule dynamics, accumulates in SCA2 cells as a consequence of defective autophagic degradation and contributes to mTORC1 hyperactivation, establishing a feed-forward loop between stress granules and impaired autophagy [49,50]. In parallel, ATXN2 aggregates were shown to sequester the mTORC1 component Raptor through their N-terminal region, suppressing mTORC1 activity and inducing compensatory autophagy [51]. Moreover, phosphoproteomic analysis of Atxn2-CAG100 knock-in mice revealed widespread alterations in selective autophagy regulators, including SQSTM1/p62-, OPTN-, TAX1BP1-, and TBK1-dependent pathways, suggesting that chronic activation and dysregulation of the autophagic machinery represent a central adaptive response to mutant ATXN2 aggregation. Together, these findings indicate that autophagy alterations in SCA2 extend beyond defective aggregate clearance and involve complex interactions between RNA metabolism, stress granules, and mTOR signaling [52].

The gene *CACNA1A* encodes for the alpha1A subunit of the P/Q voltage-dependent calcium channel, and CAG repeat expansion in this gene leads to the onset of spinocerebellar ataxia 6 (SCA6). Due to progressive oxidative stress, mitochondrial dysfunctions in cerebellar Purkinje cells were observed. However, co-localization of mitochondrial marker CoxIV and lysosomal marker Lamp1 in Purkinje cells was found to decline over disease progression; also, LC3B puncta were reduced not only in Purkinje cells, but also in the entire cellular and molecular layers of the cerebellum of SCA6 mouse models, suggesting that defective mitochondria could not be removed due to impairments in mitophagy, the selective autophagy pathway for mitochondria clearance [53]. Defects in mitophagy were previously detected also in an in vitro model of HD, where the presence of the polyQ tract in mutant Htt inhibited its mitophagy-related functions, leading to defective mitochondria accumulation and an increase in oxidative stress, which together contribute to the ongoing neurodegeneration [54].

### 2.3. SCA1, SCA7, and SCA17

Fewer studies have directly interrogated autophagy in other polyQ ataxias, such as SCA1, SCA7, and SCA17, probably due to the nuclear localization of aggregates in these kinds of spinocerebellar ataxias associated to coding repeat expansions in ataxin-1 (*ATXN1*), ataxin-7 (*ATXN7*), and TATA-box binding protein (*TBP*) genes, respectively. As a cytoplasmic process, autophagy is more likely to be involved in the previously discussed SCAs (SCA2 and SCA3) or in HD and DRPLA, where protein aggregates are shared between the nucleus and cytoplasm [55]. Nevertheless, broader explorations of polyQ disease biology suggest conserved involvement of degradative pathways. In SCA7, evidence from patient tissues, mouse models, and cellular systems supports a direct involvement of the autophagy-lysosome pathway. Increased LC3 and the lysosomal marker LAMP1 have been detected in the cerebellum of SCA7 patients, but not in the striatum, a brain area usually unaffected by SCA7, suggesting region-specific autophagy-lysosome alterations. Similar changes were observed in SCA7 knock-in mice expressing mutant ATXN7 with 266 CAG repeats, in which several lysosomal markers and LC3B were elevated in the cerebellum, while Beclin-1 and SQSTM1/p62 were sequestered into polyQ inclusions. In parallel, reduced levels of the Atg12-Atg5 complex and the LC3-lipidating enzyme Atg7 suggested impaired autophagosome biogenesis and/or maturation [56]. Mechanistically, mutant ATXN7 has been proposed to inhibit autophagy initiation through a p53-dependent mechanism. In inducible SCA7 cellular models, mutant ATXN7 increases cytoplasmic p53 and promotes its interaction with FIP200, a core component of the ULK1 autophagy-initiating complex. This favors FIP200 aggregation and destabilization of ULK1, thereby disrupting the FIP200-ULK1 complex and reducing autophagic flux. Consistently, pharmacological inhibition of p53 with pifithrin-α or disruption of the p53–FIP200 interaction restored FIP200 and ULK1 levels, improved LC3-II responses to autophagy inhibitor Bafilomycin A1, and increased cell viability, indicating that impaired autophagy contributes functionally to mutant ATXN7 toxicity [57]. Accordingly, pharmacological activation of autophagy has shown beneficial effects: rapamycin delivered through poly(lactic-co-glycolic acid) nanoparticles promoted clearance of pathological ATXN7 aggregates in ATXN7Q64-inducible neurons [58]. Among autophagy-inducing compounds, Ding and colleagues [59] identified the Hsp90 inhibitor BIIB021 as a potential therapeutic candidate for SCA1. Treatment with BIIB021 promoted clearance of ATXN1 aggregates in GFP-ATXN1[84Q]-expressing N2a cells, accompanied by reduced SQSTM1/p62 levels and restoration of LC3 dynamics, consistent with enhanced autophagic flux. Mechanistically, BIIB021 was proposed to act through activation of heat-shock factor 1 (HSF1), which upregulates autophagy-related genes, including *ATG7*, and promotes degradation of mutant ATXN1 through both autophagic and proteasomal pathways. Accordingly, two small-molecule autophagy enhancers, AUTEN-67 and AUTEN-99, improved climbing performance and extended lifespan in *Drosophila* models expressing human ATXN1Q82. AUTEN-67, which acts by inhibiting the autophagy suppressor MTMR14/EDTP, also reduced ubiquitinated protein accumulation and autophagy substrate levels, indicating improved proteostasis. Their beneficial effects were observed in neuronal populations relevant to disease pathogenesis, and autophagy-enhancing activity has also been confirmed in mammalian systems [60], further supporting autophagy as a promising therapeutic target in SCA1. While these pharmacological studies largely support a protective role for autophagy enhancement, findings from *Drosophila* models indicate that the contribution of autophagy to SCA1 pathogenesis is more complex. The *Drosophila* TFEB ortholog Mitf, a master regulator of lysosomal biogenesis and autophagy, promotes clearance of expanded ATXN1 and reduces aggregate burden, supporting a direct contribution of the lysosomal-autophagy pathway to mutant ATXN1 turnover [61]. Furthermore, mutant ATXN1 expression induces accumulation of ubiquitinated proteins together with alterations in autophagy markers, suggesting that SCA1 may be associated with a state of autophagic stress in which activation of the pathway is insufficient to efficiently resolve proteotoxic burden. Supporting this interpretation, GLaz, a *Drosophila* Lipocalin induced as part of the endogenous response to neurodegeneration, reduces ubiquitinated protein accumulation, decreases Atg8/LC3 and p62 levels, and ameliorates retinal degeneration in SCA1 flies. Interestingly, genetic inhibition of selective autophagy through downregulation of Atg8a, p62, Atg1/ULK1, or Atg4a produced a similar degree of neuroprotection and hampered the beneficial effects of GLaz, suggesting that excessive or dysregulated autophagy may itself contribute to neuronal dysfunction [62]. These findings support a model in which efficient regulation and completion of autophagic flux, rather than simple pathway activation, are critical determinants of neuronal survival in SCA1, In this context, Lee and colleagues [63] selected trehalose analogs lactulose and melibiose to treat HEK293 and SHSY5Y cells transfected with TBP/Q79-GFP, a cellular model of SCA17. Both compounds reduced insoluble TBP aggregates and increased LC3-positive puncta, indicating activation of autophagy. Importantly, their anti-aggregation effect was attenuated by the autophagy inhibitor Bafilomycin A1, supporting an autophagy-dependent mechanism of action. Consistently, prolonged administration of lactulose and melibiose reduced TBP aggregation in cerebellar slice cultures and in the cerebellum of SCA17 transgenic mice [63]. Additional support for a protective role of autophagy in SCA17 comes from studies using granulocyte colony-stimulating factor (G-CSF), which improved motor performance and reduced Purkinje cell pathology when administered during the presymptomatic stage of disease. These effects were accompanied by increased Beclin-1 and LC3-II levels, enhanced Hsp70 expression, and reduced accumulation of insoluble mutant TBP, suggesting that stimulation of autophagic clearance may contribute to neuroprotection [64].

**Figure 2:**
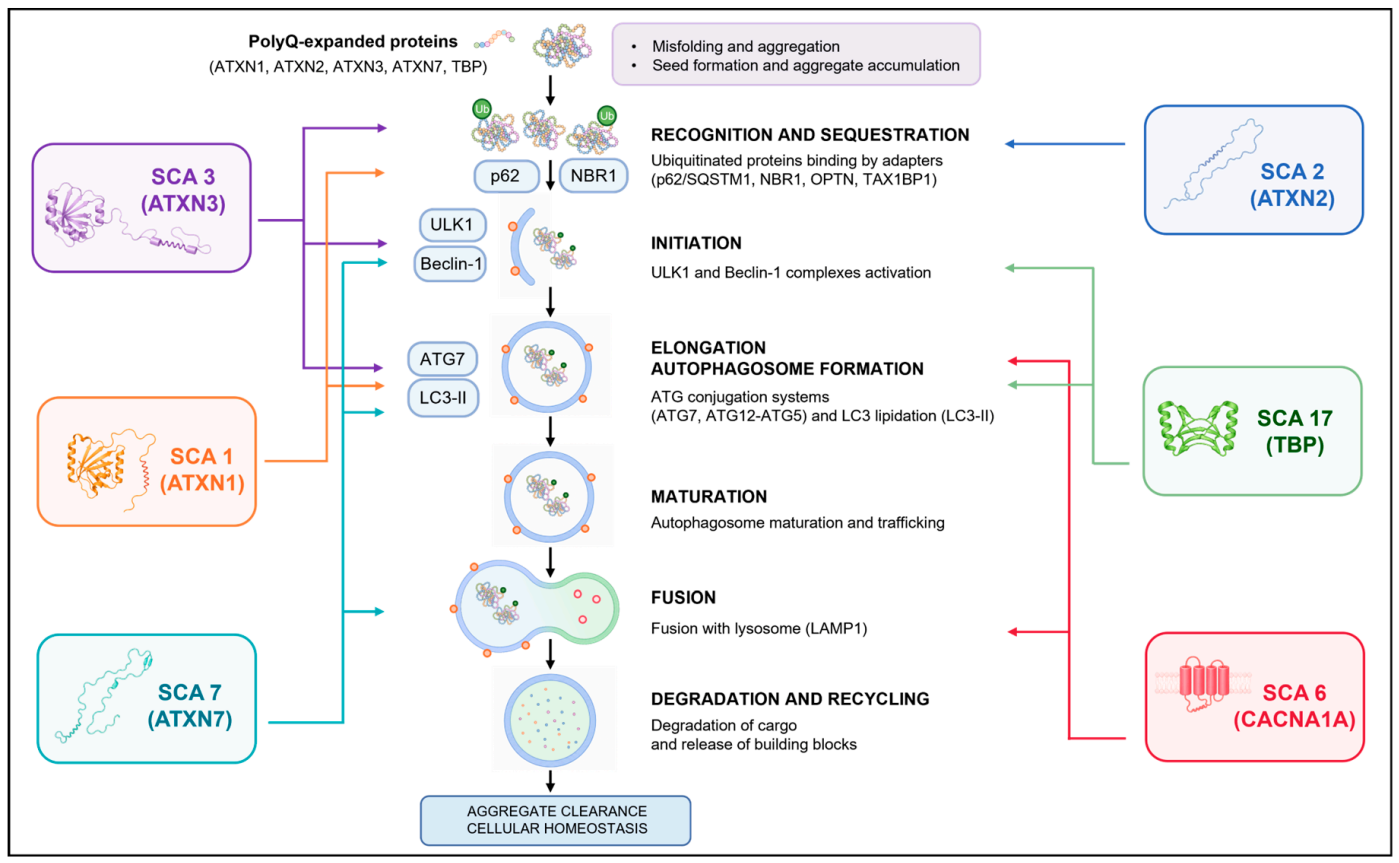
**Autophagy dysfunction in polyglutamine spinocerebellar ataxias.** Schematic representation of the autophagy pathway and the main alterations reported in polyglutamine spinocerebellar ataxias (SCAs). Polyglutamine-expanded proteins undergo misfolding and aggregation and are recognized by selective autophagy receptors, including SQSTM1/p62 and NBR1, for sequestration into autophagosomes and subsequent lysosomal degradation. Evidence from different polyQ SCAs indicates that disease-associated proteins can perturb the autophagy pathway at multiple levels, including initiation and nucleation (ULK and Beclin-1), autophagosome formation and LC3 lipidation (ATG7 and LC3-II), autophagic flux, and selective clearance of damaged organelles, such as mitochondria in the case of SCA6. The SCAs shown around the pathway identify the autophagic steps or processes reported to be altered in each disease, as indicated by solid arrows and should not be interpreted as exclusive or disease-specific sites of dysfunction. Collectively, these alterations may compromise aggregate clearance and cellular homeostasis, contributing to the progressive accumulation of toxic polyglutamine species and neurodegeneration.

Overall, although further investigation is required, accumulating evidence indicates that expanded proteins containing polyQ stretches due to CAG aberrant repetitions frequently sequester components of the autophagic machinery, disrupting autophagy initiation complexes or preventing autophagosomes’ formation, thus overwhelming protein quality control systems, collectively contributing to neuronal stress and degeneration. These pathological mechanistic insights, drawn from both molecular studies and disease models, indicate that autophagy impairment may be a shared feature across polyQ SCAs and related polyQ neurodegenerative diseases (Figure 2).

## 3. Autophagy Dysregulation in Non-Coding Repeat Expansion Ataxias: From Protein Loss-of-Function to RNA Toxicity

Non-coding repeat expansion disorders encompass a group of neurodegenerative diseases caused by the abnormal expansion of nucleotide repeats within non-coding regions of the genome. These expansions can occur in introns or untranslated regions (5′ or 3′ UTRs). In contrast to polyQ diseases, typically driven by trinucleotide repeat expansions, non-coding expansions display greater sequence variability, ranging from tri- to hexa-nucleotide repeats and often characterized by a high GC content. Despite this variability, non-coding repeat expansion disorders share a broad spectrum of clinical manifestations, including cerebellar ataxia, motor neuron involvement, cognitive impairment, and extra-neuronal features. Representative disorders include several spinocerebellar ataxias (SCA8, 10, 12, 31, 36, and 37), Friedreich’s ataxia (FA), amyotrophic lateral sclerosis/frontotemporal dementia (ALS/FTD), and myotonic dystrophy. Although arising from a common class of mutation, these diseases are driven by multiple, non-mutually exclusive molecular mechanisms, whose relative contributions to disease pathogenesis remain incompletely defined across all disorders. Repeat expansions can disrupt transcriptional regulation or enhance mRNA degradation, resulting in loss-of-function of the encoded protein. Alternatively, they can exert toxic effects at the RNA level. RNA toxicity may occur through direct sequestration of RNA-binding proteins by expanded repeat transcripts, leading to the formation of nuclear RNA foci and cytoplasmic stress granules, or indirectly via repeat-associated non-AUG (RAN) translation, which generates toxic peptides independently of canonical start codons. While RNA foci are predominantly intranuclear and observed in both neurons and glial cells, stress granules and RAN-derived proteins tend to accumulate as cytoplasmic aggregates, primarily in neurons [65].

Given the aggregation-prone nature of both expanded RNAs and their RAN translation products, autophagy is increasingly being investigated as a mechanism contributing to RNA homeostasis. In *Drosophila*, expression of double-stranded repeat RNA (rCAG·rCUG∼100) induces RNA toxicity associated with the generation of shorter single-stranded (rCAG)_7_ fragments, which have also been detected in the brains of HD patients. Notably, genetic inhibition of autophagy through silencing of *Atg1* or *Atg18* exacerbates the toxic phenotype, whereas control flies remain unaffected [66], thus supporting a functional role for autophagy in modulating RNA-mediated toxicity.

### 3.1. Frataxin Deficiency, Mitophagy Dysfunction, and Autophagy Alterations in Friedreich’s Ataxia

The homozygous GAA repeat expansion in the first intron of the *frataxin* (*FXN*) gene is known to promote histone deacetylation and, therefore, induce a conformational change in DNA that inhibits frataxin transcription, dramatically decreasing its mRNA and protein levels, ultimately leading to the onset of Friedreich’s ataxia (FA). Although its role is still not fully characterized, FXN localizes in mitochondria, where it plays a key role in iron metabolism, in particular iron–sulfur cluster and heme biogenesis. Therefore, defects in these processes caused by FXN deficiency will not only affect mitochondria but also alter cellular iron homeostasis, leading to iron accumulation and oxidative stress [67]. In physiological conditions, defective mitochondria are removed by mitophagy (mitochondria-selective autophagy), but in pathological conditions this mechanism may be interrupted and damaged mitochondria not degraded, thereby exacerbating disease progression. Indeed, mouse models for a heart-selective frataxin knockout demonstrate that, although the LC3-II/I ratio remains intact and general autophagy seems to function correctly, protein levels of both the autophagy receptor SQSTM1/p62 and mitophagy-selective E3 ubiquitin ligase Parkin (recruited to damaged mitochondria) are significantly increased. Also, FXN deficiency was able to promote mTORC1 phosphorylation, which in turn inhibits TFEB nuclear translocation by phosphorylation, ultimately reducing the autophagy rate. Key regulators of lysosomal acidification and homeostasis, as well as lysosome-associated membrane protein 1 and 2 (LAMP1 and LAMP2), were found upregulated, while lysosomal proteases cathepsins were not able to complete their maturation. Altogether, these findings suggest a dysfunctional accumulation of lysosomes, which could be responsible for a defective clearance of damaged mitochondria in frataxin-deficient mice [68]. LAMP1 overall and glycosylated levels were found increased also in FA patient-derived microglia (iMGs). Increased lysosomal density was confirmed using the lysosome-specific dye Lysotracker, although the pH-dependent dye Lysosensor demonstrated an impairment in lysosomal acidification, suggesting a dysregulation in mitochondria–lysosome interplay in FA iMGs. Consistently, when a pH-sensitive dye was used to assess mitophagy (MD1-10) under both basal and mitochondria stress conditions, authors observed reduced co-localization between mitochondria and lysosomes in FA iMGs, indicating decreased capacity in inducing mitophagy, which may account for the observed round mitochondria accumulation [69]. However, contrasting results were obtained by Edenharter and colleagues [70] in frataxin-deficient flies. Although SQSTM1/p62 protein was accumulated both in glia and muscle cells of FA flies, it also co-localized with ATG8 and LAMP1 (autophagy and lysosomal markers, respectively), indicating a correct recruitment of factors at the autophagosome. Moreover, the authors exploited the mitochondrial pH-sensitive reporter mtRosella to elegantly demonstrate in vivo that damaged mitochondria were properly tagged by both GFP and dsRed fluorescent protein, engulfed by autophagosomes and ultimately degraded by lysosomes, where the GFP signal was lost due to the acidic pH, similarly to control flies. These results suggest that, despite SQSTM1/p62 accumulation, mitophagy is not impaired in frataxin-deficient *Dropsophila*; however, it could be argued that the ordinary mitophagy flux may not be sufficient to efficiently clear damaged mitochondria [70]. Sustaining this hypothesis, the same mtRosella reporter was used in a frataxin-RNAi model of *C. elegans*, where the GFP/dsRed ratio was significantly reduced with respect to control animals, accounting for mitophagy hyperactivation. Interestingly, *FXN* silencing was also able to trigger mitophagy in vitro in a mammalian cell line: the increase in LC3-II levels upon *FXN* shRNA treatment was associated to a decrease in mitochondrial proteins superoxide dismutase (MnSOD) and Translocase of Outer Mitochondrial Membrane 20 (TOM20), and Parkin was observed to translocate from the cytoplasm to the nucleus, which signals mitochondria targeting to the autophagic machinery [71]. Enhancement of mitophagy flux was recently assessed in vivo also in a conditional *frataxin*-knockout (KO) mouse model, where the authors treated mice with autophagy inhibitor Bafilomycin A1 (BAF) and found that the LC3-II/I ratio was increased, as expected, but was higher when compared to control mice. SQSTM1/p62 and mitophagy substrate Mitofusin1 (Mfn1) accumulation was also more significant in BAF KO mice with respect to their control littermates, collectively indicating that FXN depletion induces mitophagy. However, observed accumulation of mitophagy factors Pink1 and Parkin in KO mice basal conditions would suggest an impairment of the mitophagy flux [72]. The reasons for these apparently contradictory observations remain unclear but are likely linked to differences in experimental models and methodologies used. Indeed, studies reporting impaired mitophagy were primarily conducted in cardiac tissue or patient-derived microglia and were supported by evidence of lysosomal dysfunction, defective acidification, or reduced mitochondria–lysosome interactions, whereas studies supporting mitophagy activation employed dynamic approaches, including mtRosella-based reporters or autophagic flux assays with lysosomal inhibitors. Collectively, these findings raise the possibility that frataxin deficiency initially triggers compensatory mitophagy in response to mitochondrial damage, whereas chronic mitochondrial stress and lysosomal dysfunction may progressively compromise the efficiency of mitochondrial clearance. Further studies using standardized flux measurements across different tissues and disease stages will be required to clarify these apparently contradictory findings. Nevertheless, it is clear that mitochondria turnover plays a key role in the pathogenesis of Friedreich’s ataxia and, therefore, mitophagy modulators could become interesting from a clinical perspective.

### 3.2. RNA Toxicity, RAN Translation, and Autophagy Dysfunction in Non-Coding Ataxias

Although the contribution of RNA toxicity-dependent mechanisms to autophagy dysfunction has not yet been sufficiently investigated in SCAs, growing evidence linking several SCA forms to toxic processes driven by expanded RNAs, including RNA foci, RBP sequestration, and RAN translation, suggests that these mechanisms may play an important role in autophagy dysregulation, warranting future studies to clarify how autophagic pathways contribute to RNA-mediated pathogenesis. Consistent with this emerging view, recent studies have begun to delineate mechanistic links between expanded RNA toxicity, altered stress granule dynamics, and impaired autophagic pathways, as discussed below. Due to their mainly nuclear localization, RNA foci generated by expanded transcripts are not typically direct targets of autophagy. However, expanded RNAs may interfere with the autophagic flux, thereby contributing to cellular dysfunction. Indeed, in *Drosophila* models of amyotrophic lateral sclerosis and frontotemporal dementia (ALS/FTD) generated by overexpression of 30 GGGGCC (G4C2) hexanucleotide repeats, accumulation of Ref(2)P (SQSTM1/p62 ortholog) and Lamp1 was observed in motor neurons, alongside a reduction in Atg8 (LC3 ortholog)-positive vesicles, collectively describing an impairment in the autophagy flux. Consistently, neurodegeneration in G4C2 flies was rescued via both genetic and pharmacological approaches, including Ref(2)P knockdown and treatment with rapamycin or trehalose, supporting a central role for autophagy in RNA toxicity-associated neurodegeneration. Mechanistically, RNA repeat-driven autophagy impairment may involve defective nuclear translocation of Mitf, the *Drosophila* homolog of TFEB, which normally translocates to the nucleus upon mTORC1 inhibition to activate autophagy-related genes, in a conserved manner. In line with this, expression of MYC-tagged Mitf in human cells stably expressing polyQ-*ATXN1* (SCA1 cell model) reduces aggregate ATXN1 burden in both the cytoplasm and nucleus [61]. Similarly, HeLa cells stably expressing 47 repetitions of the G4C2 hexanucleotide retain TFEB in the cytoplasm even under starvation conditions, thereby limiting autophagy induction [73].

Autophagy is also closely linked to stress granule dynamics. The autophagy receptor SQSTM1/p62 is recruited to cytosolic stress granules under stress conditions to mediate their clearance. When not efficiently removed, chronic stress granules can persist and transition from a liquid-like to a more solid, less dynamic state, promoting sequestration of RNA-binding proteins (RBPs) and contributing to cellular toxicity [74]. Among these RBPs, Staufen1 and Staufen2 (STAU1/2) are multifunctional proteins involved in RNA metabolism and recruited to stress granules to weaken their assembly. As discussed in the SCA2 section, STAU1 accumulation has been linked to impaired autophagic degradation and altered mTORC1 signaling, illustrating the existence of a bidirectional interplay between stress granules and autophagy. Similar reciprocal interactions between RBPs and autophagic pathways have been described in ALS/FTD models, where *STAU1* downregulation reduces mTORC1 activity and restores autophagic flux, whereas pharmacological inhibition of mTORC1 with rapamycin stabilizes STAU1 levels [50]. Collectively, these findings support the notion that defective stress granule turnover and impaired autophagy may establish self-reinforcing pathogenic loops that contribute to neurodegeneration.

Spontaneous formation of stress granules is further promoted by polypeptide repeats (PPRs) generated through RAN translation, likely via exacerbation of ER stress pathways. RAN peptides represent a hallmark of RNA toxicity in repeat expansion disorders and contribute to proteotoxic stress by forming SQSTM1/p62-positive cytoplasmic inclusions. In mouse models expressing dipeptide repeats (DPRs) derived from *C9Orf72* ALS/FTD expansion, these aggregates sequester components of the nuclear pore complex, thereby hindering TFEB nuclear translocation and consequent transcription of genes related to autophagosome and lysosomal biogenesis [75]. Consequently, upstream autophagy defects in repeat expansion disorders might fail to efficiently clear DPR aggregates, which in turn exacerbate autophagic dysfunction, establishing a detrimental feedback loop. CGG repeat expansions in the 5′UTR regions of different genes are the leading cause of several neurodegenerative and muscular diseases, including neuronal intranuclear inclusion disease and oculopharyngodistal myopathy. In HEK293 cells expressing 50 Flag/GFP tagged-CGG repeats in exon1 of the *GIPC1* gene, RAN translation produces polyglycine (polyGly) peptides even under conditions of canonical translation inhibition. These polyGly peptides form aggregates that co-localize with the autophagy receptors SQSTM1/p62 and TOLLIP [76]. Importantly, this interaction is not merely associative. Indeed, proteomic and functional analyses demonstrated that polyGly aggregates sequester SQSTM1/p62, TOLLIP, and additional autophagy receptors into insoluble inclusions, impairing their ability to recruit LC3 and promote autophagosome formation. As a consequence, autophagic flux is reduced, supporting a model in which RAN-derived polyGly peptides directly contribute to autophagy dysfunction by trapping essential components of the autophagic machinery rather than simply serving as substrates for degradation.

**Figure 3:**
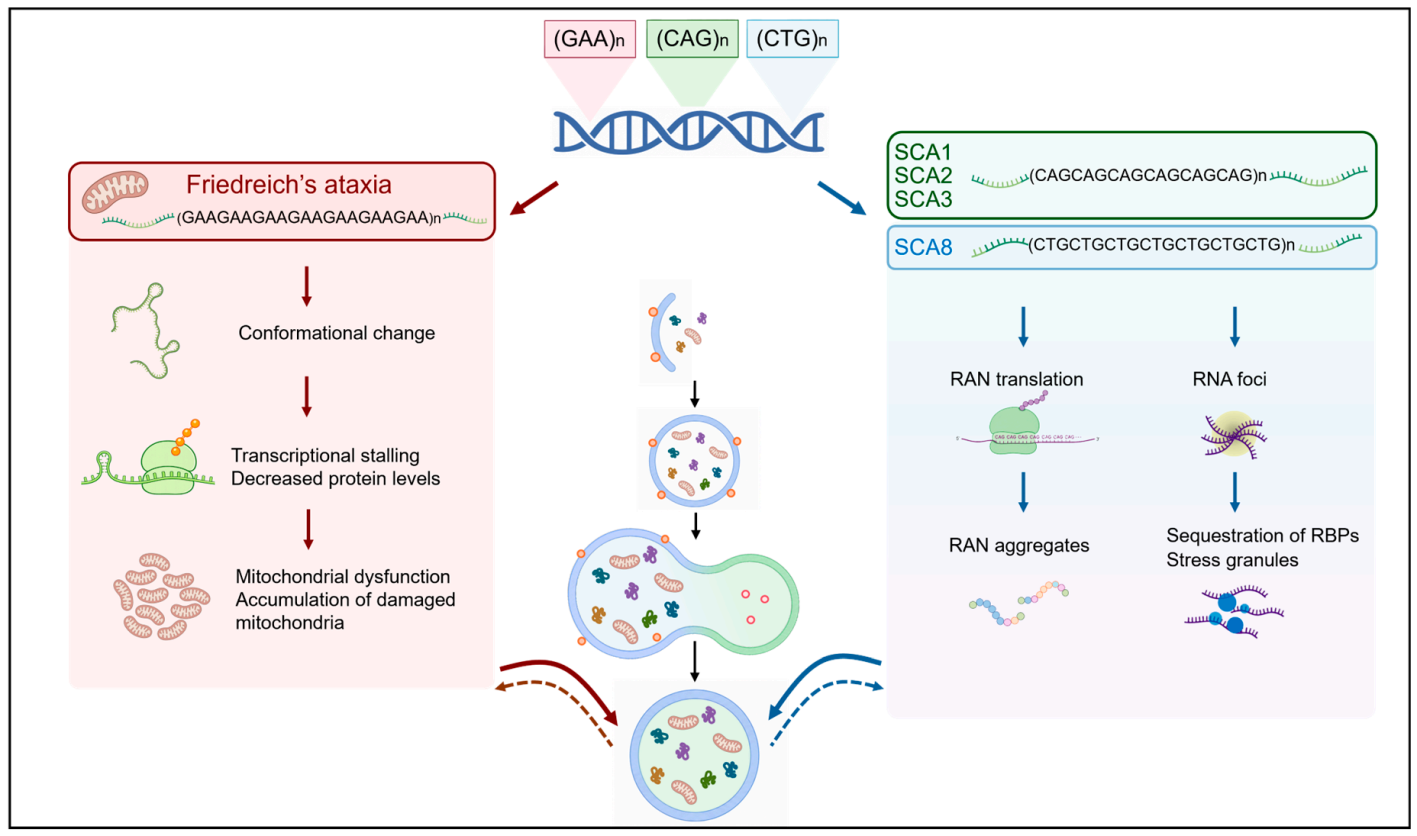
**Autophagy dysfunction in non-coding repeat expansion ataxias.** Schematic representation of the main mechanisms linking non-coding repeat expansions to autophagy dysfunction. In Friedreich’s ataxia, intronic GAA repeat expansions in the *FXN* gene induce transcriptional repression and frataxin deficiency, leading to mitochondrial dysfunction and accumulation of damaged mitochondria. Alterations in mitophagy and lysosomal function may compromise mitochondrial clearance, although the direction and extent of mitophagy dysregulation vary across experimental models. In repeat expansion ataxias associated with RNA toxicity and RAN translation, including SCA8 and polyglutamine SCAs, expanded repeat-containing RNAs may form RNA foci, sequester RNA-binding proteins, and promote stress granule formation, while RAN translation generates aggregation-prone peptides that contribute to proteotoxic stress and may interfere with autophagic clearance (solid arrows). Conversely, defective autophagic clearance may further enhance mitochondrial damage and favor the persistence and accumulation of toxic RNAs, stress granules, and RAN-derived peptides, thereby establishing self-reinforcing pathogenic loops, as indicated by dashed arrows.

Although initially described in SCA8 [77] and later observed in SCA36 [78] patients and animal models, RAN translation remains incompletely characterized in other non-coding SCA subtypes and its relationship with autophagy is still poorly understood. Interestingly, homopolymeric expansion proteins produced by RAN translation have also been identified in polyQ disorders, including HD [79], as well as SCA2 and SCA3 models, where RAN peptides were observed depending on the sequences flanking the *ATXN2* or *ATXN3* repeats, respectively [80,81]. Thus, RAN translation seems to be linked to the presence of expanded repeats even independently from their intronic or exonic localization. Accordingly, Bañez-Coronel and colleagues [82] specifically detected and characterized polySerine and polyLeucine RAN peptides in brain biopsies and mouse models across multiple polyQ SCAs (SCA1, SCA2, SCA3, SCA6, and SCA7). PolySer and polyLeu peptides form cytoplasmic ubiquitin-positive round vesicle-like structures or dense aggregates when transfected in T98 cells, suggesting an impairment in protein homeostasis and autophagy alterations. Indeed, LC3-II levels were increased in susceptible T98 and N2a cells expressing polySer and polyLeu, but not in HEK293T, which are resistant to RAN peptides’ accumulation. Interestingly, by means of either polyQ-encoding RAN-permissive or polyQ-encoding RAN-negative SCA-associated minigenes, the authors also demonstrated in vitro that polyQ and RAN peptide-derived aggregates synergistically exacerbate LC3B cleavage, thereby contributing to the observed autophagic defects. While these findings are consistent with altered autophagy and autophagosome accumulation, they do not distinguish between increased autophagosome formation and impaired autophagic clearance. In particular, whether polySer and polyLeu peptides primarily represent autophagic cargo targeted for degradation, or instead act as drivers of autophagic dysfunction, remains unresolved. However, the ability of polyGly aggregates to sequester autophagy receptors and disrupt autophagosome formation, as previously described, might suggest that RAN-derived proteins may contribute to autophagic dysfunction through direct interference with the autophagic machinery, in addition to inducing proteotoxic and ER stress responses. Importantly, in T98 cells transfected with non-ATG SCA3 (CAG)_103_ minigene, the burden of polyAla and polyGly RAN peptides was reduced in a dose-dependent manner by administration of the autophagy-activating drug Metformin, along with an increase in cell viability. Similarly, in SCA8 BAC transgenic mice, Metformin was able to improve ambulatory performance, reduce RAN protein accumulation, and attenuate neuroinflammation by decreasing astrogliosis and microglial activation [83]. Although these results support a beneficial effect of Metformin on RAN-associated toxicity, the underlying mechanism remains uncertain. Indeed, given the pleiotropic actions of Metformin on AMPK-mTOR signaling, cellular metabolism, and stress responses, it is currently unclear whether the reduction in RAN peptide burden results from enhanced autophagic degradation, decreased RAN translation, or indirect effects on cellular homeostasis. Therefore, current evidence supports an association between autophagy activation and reduced RAN peptide burden but does not yet establish that RAN proteins are directly cleared through autophagy in vivo. Thus, future studies specifically monitoring autophagic flux and RAN peptide turnover will be necessary to clarify this relationship. Nevertheless, these observations clearly establish a correlation between non-coding spinocerebellar ataxias regardless of their pathogenic mechanisms (Figure 3) and further support the therapeutic potential of targeting autophagy in repeat expansion disorders.

## 4. Challenges in Therapeutic Autophagy Modulation

Despite the encouraging efficacy of autophagy-inducing compounds in preclinical models, their clinical translation in SCAs remains challenging.

To date, trehalose is the only autophagy-modulating compound that has reached clinical evaluation in patients with SCAs. This naturally occurring, non-reducing disaccharide has attracted considerable attention because of its neuroprotective activity in several neurodegenerative disease models. Nevertheless, its precise mechanism of action remains incompletely understood. While several studies suggest that trehalose indirectly suppresses mTORC1 signaling through AMPK activation or cellular stress responses, others propose that it directly promotes TFEB activation and lysosomal biogenesis [84]. In addition, its pharmacokinetic profile raises important translational concerns. Although trehalose has recently been detected within the mouse hippocampus, suggesting that it may cross the blood–brain barrier [85], orally administered trehalose is extensively hydrolyzed by intestinal trehalase, substantially reducing its bioavailability and requiring administration of high doses [86]. These limitations may have contributed to the disappointing results of a recent randomized clinical trial in SCA3 patients, in which daily administration of 100 g of trehalose for six months was safe and well tolerated but failed to improve disease severity compared with the placebo [87].

Among mTOR-dependent autophagy activators, rapamycin remains one of the best-characterized compounds. However, its clinical application is complicated by the pleiotropic functions of mTOR signaling. Besides regulating autophagy, mTOR controls protein synthesis, cellular metabolism, proliferation, and immune responses. Consequently, systemic mTORC1 inhibition may result in significant off-target effects. Rapamycin promotes differentiation of regulatory T cells at the expense of effector T cells and interferes with antigen presentation, thereby suppressing adaptive immune responses [88]. While these properties are therapeutically advantageous in transplantation medicine, where rapamycin is routinely used to prevent graft rejection, they represent a major concern for chronic neurodegenerative disorders requiring prolonged or lifelong treatment. Long-term immunosuppression may increase susceptibility to infections while simultaneously interfering with physiological immune surveillance. Moreover, the role of neuroimmune responses in SCAs remains incompletely understood. Although chronic neuroinflammation contributes to disease progression, microglia and other immune cells also participate in tissue homeostasis and aggregate clearance. Therefore, indiscriminate suppression of immune signaling could inadvertently compromise protective mechanisms operating within the central nervous system.

Repurposing of Metformin represents another attractive therapeutic strategy owing to its established clinical safety profile and ability to activate autophagy through AMPK signaling. Nevertheless, several pharmacological aspects remain unresolved. Experimental studies indicate that Metformin crosses the blood–brain barrier through organic cation transporters, yet its distribution within the CNS appears heterogeneous. Acute administration preferentially results in cerebellar accumulation, whereas chronic treatment leads to higher concentrations in the hippocampus, frontal cortex, and pituitary gland, raising questions regarding sustained cerebellar drug exposure in SCAs [89]. Furthermore, prolonged Metformin administration is associated with vitamin B12 deficiency, a potentially relevant adverse effect in patients with SCAs, where additional spinal cord dysfunction could exacerbate pyramidal signs and gait impairment.

Several additional autophagy-modulating compounds, including cordycepin, lithium, valproate, and synthetic trehalose analogs, have shown promising effects in experimental models. However, their mechanisms of action remain incompletely characterized, and evidence supporting their efficacy in humans is still lacking. Moreover, discrepancies between experimental models further complicate clinical translation. For example, lithium-induced autophagy improves disease phenotypes in *Drosophila* models of SCA3 but fails to produce comparable benefits in murine models, suggesting species-specific or cell-type-specific differences in autophagy regulation [90]. Likewise, the pleiotropic nature of compounds, such as lithium and valproate, may affect multiple signaling pathways beyond autophagy, including Wnt signaling, making dose optimization particularly challenging [91,92]. In addition, variability in the expression of autophagy biomarkers, including SQSTM1/p62 and LC3B, further complicates the identification of universally effective therapeutic regimens.

The successful therapeutic exploitation of autophagy modulation in SCAs is likely constrained by a narrow therapeutic window, reflecting the progressive nature of autophagy dysfunction throughout disease progression. In advanced stages, the accumulation of polyglutamine aggregates may actively interfere with autophagy initiation and lysosomal function [30], potentially limiting the efficacy of pharmacological interventions that rely on an intact degradative machinery. Consequently, strategies aimed at enhancing autophagy may be most effective during early disease stages, before irreversible impairment of the autophagic flux has occurred. However, successful therapeutic modulation of autophagy requires precise regulation of the pathway itself. Both insufficient and excessive activation may be detrimental, as sustained autophagy hyperactivation has been associated with autophagy-dependent cell death [93]. Furthermore, increasing autophagosome biogenesis does not necessarily translate into enhanced cargo degradation when lysosomal function is already compromised, a condition frequently observed during advanced stages of neurodegenerative diseases [94]. Under these circumstances, pharmacological induction of autophagy may even exacerbate the accumulation of undegraded autophagic intermediates. Collectively, these observations indicate that future therapeutic strategies should move beyond indiscriminate autophagy activation toward approaches capable of restoring autophagic flux in a spatially, temporally, and cell-type-specific manner while preserving the physiological functions of this essential homeostatic pathway.

## 5. Conclusions

**Figure 4:**
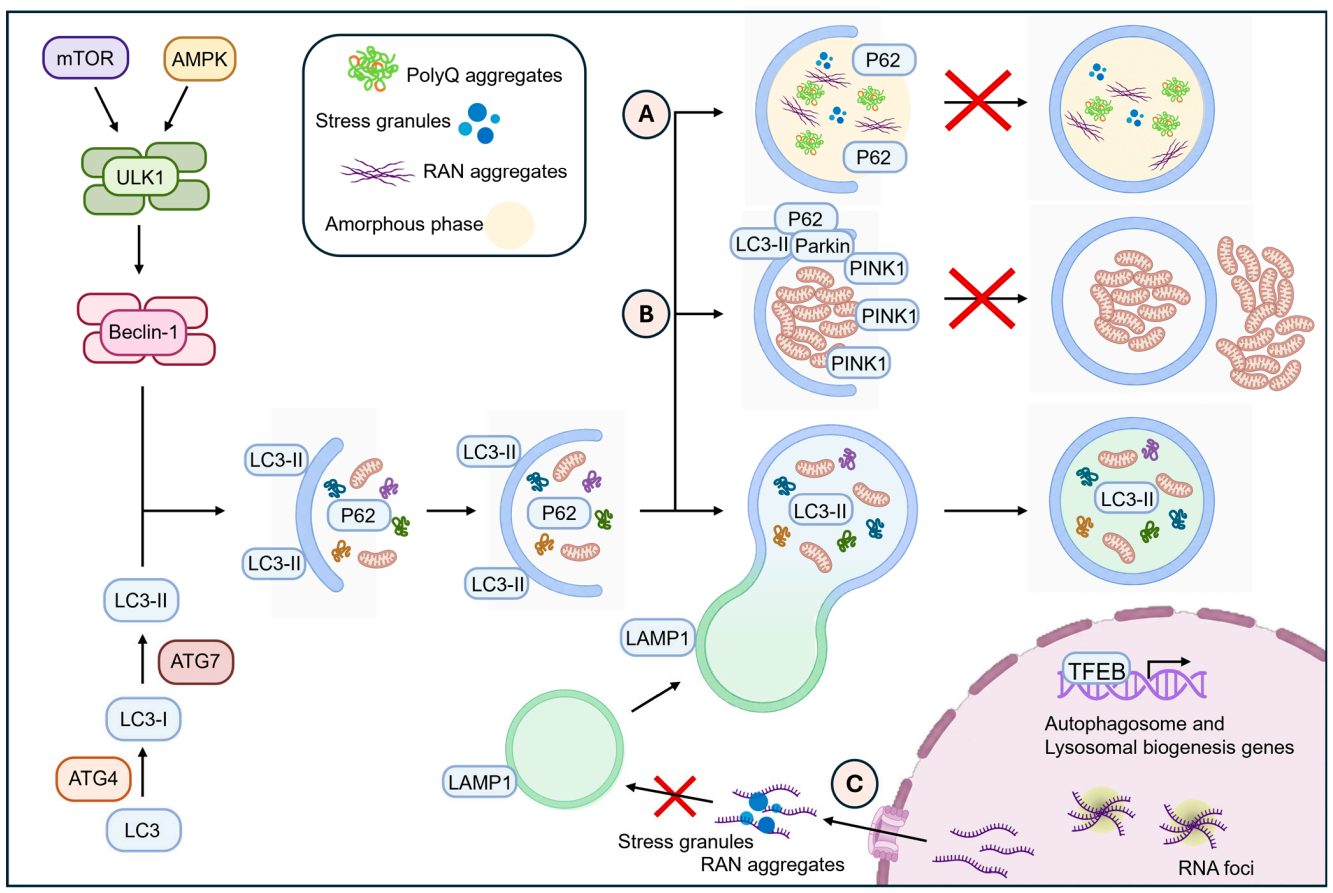
**Autophagy pathway and its impairment in repeat expansion disorders.** Schematic representation of the autophagy pathway, from initiation to cargo degradation, highlighting key regulatory components, including mTORC1, ULK1 complex, Beclin-1, LC3 processing, and lysosomal fusion. Autophagy dysfunction emerges as a recurrent pathogenic theme across repeat expansion cerebellar ataxias, with different pathogenic triggers engaging the autophagy-lysosome system through both overlapping and disease-specific routes. Under physiological conditions, cytosolic cargo is selectively recognized by autophagy receptors, such as SQSTM1/p62, sequestered into LC3-II-positive autophagosomes, and delivered to lysosomes for degradation following fusion with LAMP1-positive compartments. (**A**) Impaired clearance of protein aggregates. Aggregation-prone substrates are inefficiently recognized and/or sequestered into autophagosomes, leading to their accumulation in the cytoplasm. While amorphous phase of protein aggregates is cleared by autophagy, PolyQ aggregates, stress granules, and RAN peptides are not correctly engulfed by the autophagosome, failing their clearance. (**B**) Defective mitophagy. Damaged mitochondria fail to be properly targeted and engulfed by autophagosomes despite the involvement of mitophagy regulators, such as PINK1 and Parkin, resulting in mitochondrial accumulation and cellular stress. (**C**) Impaired RNA clearance. Expanded RNA species are not efficiently transported to or degraded within lysosomes, possibly due to defective TFEB activation and/or lysosomal dysfunctions, leading to nuclear and cytoplasmic accumulation of toxic RNA species, respectively, RNA foci and stress granules.

Collectively, evidence across coding and non-coding repeat expansion cerebellar ataxias supports autophagy dysfunction as a convergent pathogenic pathway linking proteostasis failure, RNA toxicity, and impaired organelle quality control (Figure 4). However, the available evidence does not support a single, uniform mechanism of autophagy impairment shared by all diseases. Rather, different pathogenic triggers appear to affect the autophagy-lysosome system through both overlapping and disease-specific routes. In polyglutamine SCAs and RAN translation-associated disorders, aggregation-prone proteins or peptides may directly challenge degradative pathways by impairing cargo recognition, aggregate clearance, lysosomal function, and TFEB-dependent transcriptional programs. In contrast, in Friedreich’s ataxia and RNA toxicity-driven disorders, autophagy alterations may arise from distinct upstream mechanisms, including mitochondrial dysfunction, altered cellular metabolism, RNA-binding protein dysregulation, stress granule dynamics, and impaired stress-response pathways.

Thus, autophagy should be viewed as a point of functional convergence rather than a single mechanistic endpoint identically reached in all ataxias. This distinction has therapeutic relevance, as it argues against indiscriminate autophagy activation and highlights the need to define, for each disease context, whether the main defect involves autophagosome formation, cargo recognition, lysosomal degradation, mitophagy, or transcriptional regulation of lysosomal biogenesis. Although the nature of autophagic defects appears disease- and context-dependent, studies in cellular and animal models increasingly indicate that restoring autophagy or selective autophagic pathways may mitigate repeat-driven toxicity and modify disease progression (Table 1).

An important limitation of the current literature is the frequent reliance on static autophagy markers, such as LC3-II levels, LC3 puncta, or SQSTM1/p62 accumulation, to infer autophagy dysfunction. While these measurements provide valuable information, they do not reliably distinguish between enhanced autophagosome biogenesis and impaired autophagosome clearance. Consequently, some conclusions regarding autophagy impairment in SCAs should be interpreted with caution. Future studies should prioritize direct assessment of autophagic flux through complementary approaches, including tandem fluorescent LC3 reporters, lysosomal inhibition assays, cargo degradation measurements, and live-cell imaging of autophagic dynamics. The adoption of these methodologies will be essential to define the precise nature of autophagy alterations across different cerebellar ataxias, clarify how autophagy intersects with distinct repeat-expansion-mediated insults, and support the rational development of autophagy-targeted therapeutic strategies.

## Figures and Tables

**Table 1:** **Proposed autophagic defects across repeat expansion cerebellar ataxias.** Distinct repeat expansion cerebellar ataxias appear to affect different stages of the autophagic pathway, ranging from autophagy initiation and autophagosome biogenesis to selective mitophagy, lysosomal degradation, and flux regulation. Experimental model refers to the systems in which the mechanism has been investigated, while functional rescue indicates how genetic or pharmacological restoration of autophagy ameliorates disease-associated molecular or behavioral phenotypes. Given the frequent reliance on static autophagy markers, the precise steps affected remain uncertain for some disorders. ↑, Increase; ↓, decrease; n.a., no experimental data available.

Disease	Key Evidence	Step Affected	Experimental Model	Functional Rescue
**SCA1**	Altered Atg8/LC3 and p62 levels	Flux regulation	Cells, *Drosophila*	✓ *Mitf* expression in cells ✓ *Glaz* expression in *Drosophila* ✓ BIIB021 treatment in cells ✓ AUTEN-67 and AUTEN-99 treatment in *Drosophila*
**SCA2**	SQSTM1/p62 and LC3B accumulation, impaired mTOR signalling, OPTN and TBK1 alterations	Flux regulation/cargo recognition	Cells, mice, human fibroblasts, human tissue	✓ cordycepin treatment in mice
**SCA3**	↓ Beclin-1, impaired LC3-I lipidation, SQSTM1/p62 accumulation; ↓ ULK1/2; ↓ Neuropeptide Y	Initiation/ autophagosome biogenesis	Cells, mice, zebrafish, human fibroblasts, iPSC-derived neurons	✓ Beclin-1, ULK1/2 or Neuropeptide Y restoration in mice ✓ n-BP or trehalose treatment in mice ✓ autophagy activators treatment in zebrafish
**SCA6**	↓ CoxIV-LAMP1 colocalization and LC3B puncta	Selective autophagy (mitophagy)	Cells, mice	n.a.
**SCA7**	↑ LC3, LC3B and LAMP1, Beclin-1 and p62 sequestered into PolyQ inclusions, ↑ p53-FIP200 interaction, ↓ FIP200-ULK1 complex, ↓ ATG7 and Atg12-Atg5 complex	Initiation/ autophagosome biogenesis and/or maturation	Cells, mice, human tissue	✓ pifithrin-α or rapamycin treatment in cells
**SCA17**	↓ LC3 puncta	Flux (unclear)	Cells, mice	✓ trehalose analogs treatment in cells and mice
**RAN translation-ataxias (SCA8, SCA36, polyQ SCAs)**	PolySer/polyLeu-induced LC3-II accumulation in polyQ SCAs	Flux regulation (unclear)	Cells, mice	✓ Metformin treatment in SCA3 cells and SCA8 mice
**Friedreich’s ataxia**	↑ p62, Pink1 and Parkin, ↑ mTORC1 phosphorylation, ↑ LAMP1 and LAMP2, immature cathepsins	Lysosomal acidification and homeostasis/mitophagy	Cells, flies, worms, mice, human iMGs	n.a.

## Data Availability

No new data were created or analyzed in this study. Data sharing is not applicable to this article.

## Abbreviations

The following abbreviations are used in this manuscript:

SCA	spinocerebellar ataxia
STRs	short tandem repeats
polyQ	polyglutamine
RAN	repeat-associated non-AUG
RBPs	RNA-binding proteins
RNPs	ribonucleoproteins
HD	Huntington’s disease
DRPLA	dentatorubral-pallidoluysian atrophy
SBMA	spinal and bulbar muscular atrophy
FA	Friedreich’s ataxia
CMA	chaperone-mediated autophagy
IMM	inner mitochondrial membrane
OMM	outer mitochondrial membrane
ALS/FTD	amyotrophic lateral sclerosis/frontotemporal dementia
PPRs	polypeptide repeat proteins
DPRs	dipeptide repeat proteins
iPSCs	induced pluripotent stem cells
iMGs	induced microglia
